# Editorial: Gut-liver-brain axis: a complex network influences human health and diseases

**DOI:** 10.3389/fnins.2023.1241069

**Published:** 2023-07-13

**Authors:** Hongjin Wu, Yongjian Zhang, Juehua Yu, Ming Shi

**Affiliations:** ^1^International Research Center for Regenerative Medicine, Boao International Hospital, Qionghai, China; ^2^Department of Surgery Oncology, Harbin Medical University Cancer Hospital, Harbin, China; ^3^School of Life Science and Technology, Harbin Institute of Technology, Harbin, China

**Keywords:** gut-liver-brain axis, host immune responses, gut microbiota, systemic inflammation, metabolism dysfunction

The gut-liver-brain axis consists of the complex interplay between the gut–brain, gut–liver, and liver–brain axes, and is a multidirectional communication network that links the enteric, hepatic, and central nervous systems. This communication network has been extended to involve endocrine, humoral, metabolic, and immune routes of communication. Through this network, the brain affects intestinal and hepatic activities, including the activity of innate immune cells and immune effector cells (Teratani et al., 2020). Meanwhile, the gut and liver influence cognition and mental health through the regulation of microbiota and host immune responses. Wang et al. find there was a potential causal relationship between gut microbiota and attention deficit hyperactivity disorder and suggest that the gut bacteria found in this study may reduce the occurrence of attention deficit hyperactivity disorder. Hildenbrand et al. investigate predisposing and precipitating risk factors for delirium (the most common acute neuropsychiatric syndrome in hospitalized patients), and find that delirium in precipitating gastrointestinal and hepato-pancreato-biliary diseases was not associated with higher age *per se*, but with cognitive and functional impairment. Diet is a modulator of the microbiome and is known to impact the gut-brain axis, including its influence on acute brain injuries. Krakovski et al. show that diets and probiotics beneficially modulate immune and neuronal functions, as well as the therapeutic importance of modulation by diets and probiotics.

An impaired gut-liver-brain axis in patients is associated with several diseases, such as hepatic encephalopathy, and Alzheimer's disease. The mechanisms behind these diseases are unclear although within-host evolution of gut microbiota, metabolism dysfunction, and systemic inflammation through gut dysbiosis have been proposed. Nguyen and Swain evaluate the potential mechanistic avenues within the gut-liver-brain axis that might be altered in the setting of chronic liver disease that drive the development of the symptoms related to fatigue, social withdrawal, and mood disturbances. They discuss how perturbations in host immune response, microbiome, neural responses, and metabolite composition could affect the central nervous system by analyzing both clinical and pre-clinical studies (Nguyen and Swain). In addition to the mechanism studies, methodology research that identifies the potential links between microbes and diseases will facilitate disease diagnosis and prevention. The reliable prediction of microbe-disease associations using a graph convolutional attention network has been achieved, which can be expected to predict unknown microbe-disease associations facilitating disease diagnosis and prevention (Shi et al.).

Taken together, the articles and review articles in this Research Topic reveal new insights into the gut-liver-brain axis or discuss how the gut-liver-brain axis works in human health and diseases from different levels and perspectives, making contributions to our understanding of the gut-liver-brain axis, improving therapeutic approaches for the related diseases. Hopefully, this Research Topic will motivate researchers to study the mechanisms of the gut-liver-brain axis and related diseases, from basic scientific research to clinical applications.

## Author contributions

MS wrote the editorial. HW, YZ, and JY edited and approved this editorial. All authors contributed to the article and approved the submitted version.

## References

[B1] Teratani T. Mikami Y. Nakamoto N. Suzuki T. Harada Y. Okabayashi K.. (2020). The liver-brain-gut neural arc maintains the T(reg) cell niche in the gut. Nature 585, 591–596. 10.1038/s41586-020-2425-3 32526765

